# Supplementary material for: Knowledge, Attitudes, and Practices Related to COVID-19 Among Malawi Adults: A Community-Based Survey
Source: Int J Environ Res Public Health. 2021 Apr 13;18(8):4090. doi: 10.3390/ijerph18084090 (PMC8069913; doi:10.3390/ijerph18084090)
Supplement: Supplementary file 1 [file ijerph-18-04090-s001.zip › Questionnaire.docx]

**Survey: Knowledge, Attitude, Practice of Malawi residents regarding COVID-19.**

Interviewer ID: _____ Questionnaire ID: ______ Date: _______

We designed this questionnaire to investigate knowledge, attitude, practice (KAP) on COVID-19 among general adults in Lilongwe, Malawi for better prevention and control. Your participation in this study is completely voluntary and you may refuse to participate or leave the study at any time. If you decide to not participate in the study it will not result in any penalty or loss of benefits to which you are otherwise entitled. Your ID will only be recorded to document that you have agreed to participate in this research. It will not be put in any of the project documents to be prepared from this research. Only the research team will have access to the data provided. The survey will take approximately 15 minutes to complete. Thank you for your support!

Do you agree to continue with the study?

□YES(Continue) □NO(Stop)

Have you heard of COVID-19(novel coronavirus)?

□Yes (Continue) □No (Stop)

Instruction: select the option according to your own idea. (write ”✓” on the option number)

| **K1: When did you first hear about COVID-19?**  **[1 answer option]** | 1.1 | one month ago |
| --- | --- | --- |
|  | 1.2 | within one month |
|  | 1.3 | within one week |
|  | 1.4 | today |
|  | 1.5 | don’t know |
| **K2: Where/from whom did you first hear about COVID-19?**  **[check multiple answer option]** | 2.1 | family/neighbor/friend |
|  | 2.2 | newspaper/magazine |
|  | 2.3 | television |
|  | 2.4 | radio |
|  | 2.5 | health care professional |
|  | 2.6 | internet |
|  | 2.7 | social media |
|  | 2.8 | government |
|  | 2.9 | In church/from religious leader |
|  | 2.10 | other |
| **K3: What are the signs and symptoms of COVID-19?**  **[check multiple answer option]** | 3.1 | fever |
|  | 3.2 | fatigue |
|  | 3.3 | dry cough |
|  | 3.4 | headache |
|  | 3.5 | myalgia |
|  | 3.6 | shortness of breath |
|  | 3.7 | dyspnea |
|  | 3.8 | don’t know |
| **K4: Does everybody who gets COVID-19 show symptoms?**  **[1 answer option]** | 4.1 | yes |
|  | 4.2 | no |
|  | 4.3 | don’t know |
| **K5: How does a person get COVID-19?**  **[check multiple answer option]** | 5.1 | close contact with someone infected by COVID-19 |
|  | 5.2 | talking with someone coughing and sneezing |
|  | 5.3 | sexual intercourse |
|  | 5.4 | mosquito bite |
|  | 5.5 | eating wild animals |
|  | 5.6 | touching surface of items attached by virus |
|  | 5.7 | don’t know |
| **K6: Who can get COVID-19?**  **[check multiple answer option]** | 6.1 | aged persons |
|  | 6.2 | adult men |
|  | 6.3 | adult women |
|  | 6.4 | boys |
|  | 6.5 | girls |
|  | 6.6 | pregnant women |
|  | 6.7 | health workers |
|  | 6.8 | everybody can get COVID-19 |
|  | 6.8 | don’t know |
| **K7:How can people prevent COVID-19？**  **[check multiple answer option]** | 7.1 | washing hands |
|  | 7.2 | avoiding touching your eyes, nose, and mouth with unwashed hands |
|  | 7.3 | use of disinfectants |
|  | 7.4 | herbal supplements |
|  | 7.5 | covering your cough |
|  | 7.6 | A balanced diet |
|  | 7.7 | staying home when you are sick |
|  | 7.8 | avoiding close contact with someone who is sick |
|  | 7.9 | use caution when opening mail |
|  | 7.10 | getting the flu shot |
|  | 7.11 | regular exercise |
|  | 7.12 | wearing a face mask |
|  | 7.13 | drinking alcohol |
|  | 7.14 | don’t know |
| **K8: Is there a special drug or an effective treatment for COVID-19?**  **[1 answer option]** | 8.1 | yes |
|  | 8.2 | no |
|  | 8.3 | don’t know |
| **K9: You should use antibiotic to treat COVID-19.**  **[1 answer option]** | 9.1 | yes |
|  | 9.2 | no |
|  | 9.3 | Don’t know |

| **A1: COVID-19 is an important issue/problem in your community.**  **[1 answer option]** | 1.1 | Strongly disagree |
| --- | --- | --- |
|  | 1.2 | disagree |
|  | 1.3 | neutral |
|  | 1.4 | agree |
|  | 1.5 | Strongly agree |
| **A2: I am concerned about that I would be infected by COVID-19.**  **[1 answer option]** | 2.1 | Strongly disagree |
|  | 2.2 | disagree |
|  | 2.3 | neutral |
|  | 2.4 | agree |
|  | 2.5 | Strongly agree |
| **A3: My life has been disturbed by COVID-19.**  **[1 answer option]** | 3.1 | Strongly disagree |
|  | 3.2 | disagree |
|  | 3.3 | neutral |
|  | 3.4 | agree |
|  | 3.5 | Strongly agree |
| **A4: COVID-19 would be controlled successfully.**  **[1 answer option]** | 4.1 | Strongly disagree |
|  | 4.2 | disagree |
|  | 4.3 | neutral |
|  | 4.4 | agree |
|  | 4.5 | Strongly agree |
| **A5: The measures of government are effective.**  **[1 answer option]** | 4.1 | Strongly disagree |
|  | 4.2 | disagree |
|  | 4.3 | neutral |
|  | 4.4 | agree |
|  | 4.5 | Strongly agree |
| **A6: If somebody in my family were to get COVID-19,I would want to remain private/a secret. [1 answer option]** | 6.1 | yes |
|  | 6.2 | no |
|  | 6.3 | Don’t know |
| **A7: If a person gets COVID-19, are they discriminated against or stigmatized because of it? [1 answer option]** | 7.1 | yes |
|  | 7.2 | no |
|  | 7.3 | Don’t know |
| **A8: Whose responsibility is it to prevent you/your house hold/your community from getting COVID-19?**  **[check multiple answer option]** | 8.1 | Personal responsibility(individual) |
|  | 8.2 | Household head (family) |
|  | 8.3 | Community /religious leaders |
|  | 8.4 | Health workers |
|  | 8.5 | Local government administration |
|  | 8.6 | National government |
|  | 8.7 | Local organizations |
|  | 8.8 | International organizations |
|  | 8.9 | god |
|  | 8.10 | other |
| **A9: Who can effectively treat a person with COVID-19?**  **[check multiple answer option]** | 9.1 | Pharmacy/local drug vendor |
|  | 9.2 | Public health post/health centre/health facility |
|  | 9.3 | Public hospital |
|  | 9.4 | Local healer |
|  | 9.5 | Private doctor |
|  | 9.6 | other |
| **A10: What worries or concerns you most about COVID-19?**  **[check multiple answer option]** | 10.1 | It can make me sick |
|  | 10.2 | It can cause economy downturn |
|  | 10.3 | It can hinder social activities |
|  | 10.4 | other |
| **A11：If you get COVID-19, what will you do?**  **[check multiple answer option]** | 11.1 | Stay at home and do nothing |
|  | 11.2 | Stay at home and take medication to bring down the fever and drink a lot of fluids |
|  | 11.3 | Be isolated |
|  | 11.4 | Go to the health centre |
|  | 11.5 | Go to private doctor |
|  | 11.6 | Go to pharmacy |
|  | 11.7 | Speak to a community-based health worker or volunteer |
|  | 11.8 | Speak to a community leader |
|  | 11.9 | Go to a local healer |
|  | 11.10 | Go to church |
|  | 11.11 | Don’t know |

| **P1: Since you heard about COVID-19, have you taken any action to prevent yourself from getting COVID-19?** | 1.1 | yes |
| --- | --- | --- |
|  | 1.2 | No (go to P4) |
|  | 1.3 | Don’t know (go to P4) |
| **P2：If yes (for P1), what action have you taken to prevent yourself/family from getting COVID-19?**  **[check multiple answer option]** | 2.1 | Avoiding going to crowded places |
|  | 2.2 | Avoiding taking public transportation. |
|  | 2.3 | Washing hands more frequently. |
|  | 2.4 | Wearing masks when leaving home. |
|  | 2.5 | Practicing social distancing。 |
|  | 2.6 | Praying to god |
|  | 2.7 | Don’t know |
| **P3: What challenges/difficulties did you face in taking that action?**  **[check multiple answer option]** | 3.1 | Difficult to make time to take preventive measures |
|  | 3.2 | Difficult to make money and resources to take preventive measures |
|  | 3.3 | Difficult to have access to necessary items (e.g.to buy masks) |
|  | 3.4 | I had to overcome people around me who didn’t want me to take action |
|  | 3.5 | I didn’t face any challenges or difficulties |
|  | 3.6 | other |
| **P4: If NO (for P1), why have you not taken any action to prevent yourself from getting COVID-19?**  **[check multiple answer option]** | 4.1 | I am not at risk/my household is not at risk |
|  | 4.2 | I don’t think COVID-19 is a problem |
|  | 4.3 | I don’t mind if I get COVID-19 |
|  | 4.4 | Preventing COVID-19 is not a priority |
|  | 4.5 | No time |
|  | 4.6 | No resources or access |
|  | 4.7 | I think preventative measures aren’t effective |
|  | 4.8 | I don’t know how to prevent COVID-19 |
|  | 4.9 | Other people are doing what is necessary to prevent me from getting COVID-19 |
|  | 4.10 | You can’t prevent COVID-19 |
|  | 4.11 | Other |

**Demographic**

**Instruction: please select the box next to the answer of your choice.**

1. **Gender**

□male □female

1. **Age**

□18-25 □26-35 □36-45 □46-55 □55+

1. **Education**

□No education □Primary incomplete □Primary complete

□Secondary incomplete □Secondary complete □More than secondary

1. **Region**

□urban □rural

1. **Religion**

□Christianity □Islamism □other

1. **Occupation**

□Unemployed □Full-time housewife with children □Agriculture □Domestic service □manual labor□Sales and services □Clerical □Professional/Technical/Managerial

1. **Marital status**

□Married □Not married

1. **How to describe your house condition? [check multiple answer option]**

□we have electricity, and it can continue at least half a day per day

□we have clear water resource(piped into dwelling or borehole with pump or protected dug well)

□we have toilets in good condition(flush or ventilated improved latrine)

□we are not crowded(5 or fewer people per room)

□we have a firm roof(tiles or galvanized iron or concrete)

1. **which do you and your family own? [check multiple answer option]**

□radio □television □stove □fridge □sofa □mobile phone

□bicycle □car □motorbike

1. **Where do you often get the information of COVID-19? [check multiple answer option]**

□Television □Radio □Newspaper/magazine □Social media □Internet

□Health care professional □Friends/neighbors/relatives □Leaflet □Other

1. **How many persons would you contact with in one day?**

□0-5 □5-10 □10+

1. **What is the risk that you will get COVID-19 in the next 6 months?**

□Definitely □High risk □Medium risk □Low risk □No risk

**Thanks for your participation!**
